# Oculomotor anticipation reveals a multitude of learning processes underlying the serial reaction time task

**DOI:** 10.1038/s41598-021-85842-x

**Published:** 2021-03-18

**Authors:** Amir Tal, Ayala Bloch, Haggar Cohen-Dallal, Or Aviv, Simone Schwizer Ashkenazi, Moshe Bar, Eli Vakil

**Affiliations:** 1 Gonda Multidisciplinary Brain Research Center, Bar-Ilan University, Ramat-Gan, Israel; 2 Department of Psychology, Bar-Ilan University, Ramat-Gan, Israel; 3 Department of Psychology, Columbia University, New York, NY 10027 USA

**Keywords:** Cognitive neuroscience, Human behaviour

## Abstract

Sequence learning is the cognitive faculty enabling everyday skill acquisition. In the lab, it is typically measured in speed of response to sequential stimuli, whereby faster responses are taken to indicate improved anticipation. However, response speed is an indirect measure of anticipation, that can provide only limited information on underlying processes. As a result, little is known about what is learned during sequence learning, and how that unfolds over time. In this work, eye movements that occurred before targets appeared on screen in an ocular serial reaction time (O-SRT) task provided an online indication of where participants anticipated upcoming targets. When analyzed in the context of the stimuli preceding them, oculomotor anticipations revealed several simultaneous learning processes. These processes influenced each other, as learning the task grammar facilitated acquisition of the target sequence. However, they were dissociable, as the grammar was similarly learned whether a repeating sequence inhabited the task or not. Individual differences were found in how the different learning processes progressed, allowing for similar performance to be produced for different latent reasons. This study provides new insights into the processes subserving sequence learning, and a new method for high-resolution study of it.

## Introduction

A fundamental aspect of adaptive behavior is the encapsulation of useful chains of actions into one efficient procedure. Underlying this process is the ability to sequence incoming information and ongoing action, an ability termed sequence learning^1^. The prevailing assumption in the literature is that successful sequence learning results in improved anticipation of sequence elements ahead of time^2,3^. Since its introduction by Nissen and Bullemer (1987)^4^, the serial reaction time (SRT) task has become the hallmark paradigm for studying sequence learning. In the SRT task, participants become quicker to respond to sequences they were previously exposed to, indicating acquired knowledge of the sequence. Critically, response not only becomes faster with exposure to the sequence, but it also slows down when the sequence is replaced by a new sequence (an “Interference sequence”) and subsequently recovers when the original sequence is restored.

This simple task has spawned a rich literature concerning, among other topics, the explicitness^5^, mechanism^6^ and locus^7^ of the learning induced in it. However, it remains unknown what precisely is learned during sequence learning. This is in part since the SRT task relies on the speed of response, which can only attest to the success of anticipation but not to its content.

**Figure 1:**
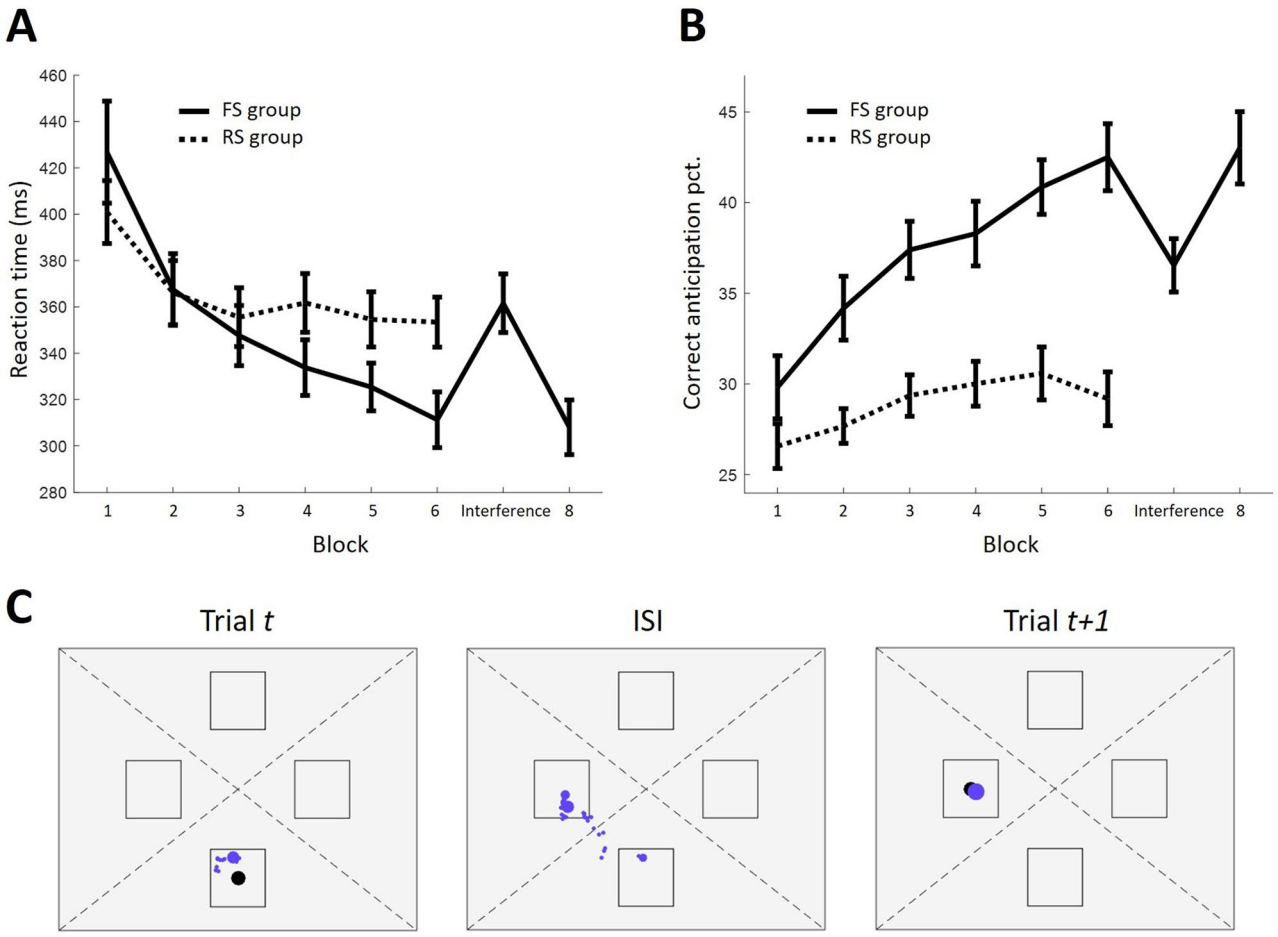
Oculomotor and motor effects of the SRT task in the FS and RS groups. (**A**) Median RT per sequence averaged per each experiment block. "Interference" indicates a different sequence governing the task in block seven. Trajectory in the FS group provides standard SRT effects of learning, interference, and recovery. RT of RS group does not reach the same speed as in the FS group. (**B**) Average percentage of correct oculomotor anticipation per experiment block mirrors the trajectory of RT. Error bars denote standard error of the mean. (**C**) Eye movements of a single participant during two trials and the ISI in between them. Blue dots mark recorded gaze location. Dot size reflects the duration of gaze, and so large dots reflect fixations. Gaze is binned into one of four regions (dotted lines), according to what target location (square) it is closest to. On trial *t*, a target (black dot) appeared at the bottom location. Fixations during the trial occurred in the same region as the target. During the ISI, fixations were detected in the bottom and then in the left regions of the screen. In the subsequent *t* + *1* trial, the target appeared in the left location, and fixations followed it. The measure of interest in this work is the location of fixations during ISIs. This is considered the oculomotor anticipation. In this case, the fixation during the ISI is regarded a correct anticipation, as the last fixation during the ISI was in the region in which the next target appeared in.

Recently, we have developed an ocular version of the SRT task (O-SRT^8^). In the O-SRT task, the spatial layout and temporal dynamics of the task allow detecting eye movements to target locations before targets appear on screen. The standard effects of the SRT task are reproduced in the O-SRT task both when the task is activated by fixation on the target (oculomotor activation version) and when it is activated by standard manual response to the target (manual activation version). In the latter version, even though participants provide manual responses to target locations, the learning, interference and recovery effects found in reaction time (RT) can also be found in the rate in which participants fixate on correct target locations ahead of time (Fig. 1). These effects stem from eye movements made during inter-stimulus intervals (ISIs)—*before* stimuli had appeared—and so, in contrast to RT, gauge anticipation directly. Moreover, they inform on what participants had anticipated, and not only whether anticipation was correct or not. In the current work, we sought to utilize this new direct measure of anticipation to uncover the processes underlying the evolvement of sequence learning. We have conducted a novel analysis of the oculomotor signal obtained in the manual activation O-SRT task, focusing on *incorrect* anticipations.

Using the immediately preceding pair of targets and the sequence governing the stimuli, semantics can be granted to anticipation attempts made by each participant at each trial^9^. As there are four possible target locations in our task, three fixation locations are incorrect at any given trial. These three are incorrect for different reasons, and we show that production of them is highly revealing of the knowledge held by participants throughout the task. Specifically, we find that knowledge of the statistical rules governing the task is acquired in addition to knowledge of the task sequence itself.

In order to isolate sequence learning from other knowledge acquired in the task we have collected data under two different conditions. Both conditions share the same statistical rules, but in one condition there is a fixed sequence embedded in the task (fixed-sequence group; FS), and in the other there is no repeating sequence embedded in the task (a pseudo-random stream of stimuli, referred to hereafter as random-sequence group; RS). It should be noted that in this analysis eye movements during ISIs are regarded responses to the stimuli that precede them^3^. Because this method reveals learning that is contingent not on the target stimulus but rather on the stimulus that precedes it, and it relies on a natural response (shift of gaze) that is not the one acquired in the task, we believe our results favor an interpretation that learning of stimulus-stimulus contingencies had occurred. However, whether oculomotor learning indeed reflects perceptual learning, in opposed to motor or S-R rule learning^7^, is not the focus of the current work. Rather, we wish to focus here on the kind of knowledge that is reflected in these oculomotor responses.

We begin this report with analysis of anticipations according to their compliance with the statistical constraints characterizing the task (the task grammar). We show that both groups learn the grammatical rules shared across their tasks in a similar manner, consistent with the hypothesis that this is a different learning process than sequence learning in the SRT task. Next, we perform an in-depth analysis of all four types of anticipations available in the task (two grammatical and two ungrammatical), and their relation to RT. We first follow the evolvement of the Main anticipation signal, corresponding to correct anticipation and the sequence learning process which is the target of the standard SRT task. We then describe the two incorrect shifts of gaze, one of which is grammatical and the other not. Then, we follow the last fixation type, which is also incorrect but does not entail a shift in gaze, and hence represents no attempt of anticipation to begin with. We conclude this report by analyzing the RT signal as parsed according to fixation type. Discussion and references to relevant literature are given in conjunction with the results.

## Results and discussion

Fifty-nine participants were recruited for an O-SRT task in which targets could appear at one of four possible locations on screen and a corresponding keyboard response was required. Twenty-nine participants comprised the FS group. Their task was made of eight blocks: six blocks governed by a fixed sequence (Main sequence), followed by one block of a different sequence (Interference sequence), and one final block of the original Main sequence. The first six blocks are referred to as the learning blocks, the seventh block the Interference block and the last block the Recovery block. Thirty other participants comprised the RS group. In the RS task, target order obeyed the same statistical constraints as in the FS task, but there was no repeating sequence embedded in the task. There is therefore no sequence interference in the RS task, and it comprised six blocks only (see *procedure* for details on how the RS task was generated). Importantly, neither group was given any information on the structure of the stimuli stream nor told that any structure exists.

### Grammar learning

Both groups of participants in our study were exposed to similar regularities in the transition of target locations from one trial to the other. Specifically, targets never appeared in the same location of the last target, and only seldom appeared in the location of the target before last. These regularities constitute the grammar of the task. They do not determine what location will appear next, but they do limit the possibilities. It is hence knowledge that can be acquired separately from sequence learning. We therefore first examined how eye movements during the task complied with the task grammar. Fixations during ISIs were considered ungrammatical if they were at the location of the target in the previous or the before-previous trial, and grammatical if they were at either of the other two possible locations.

**Figure 2:**
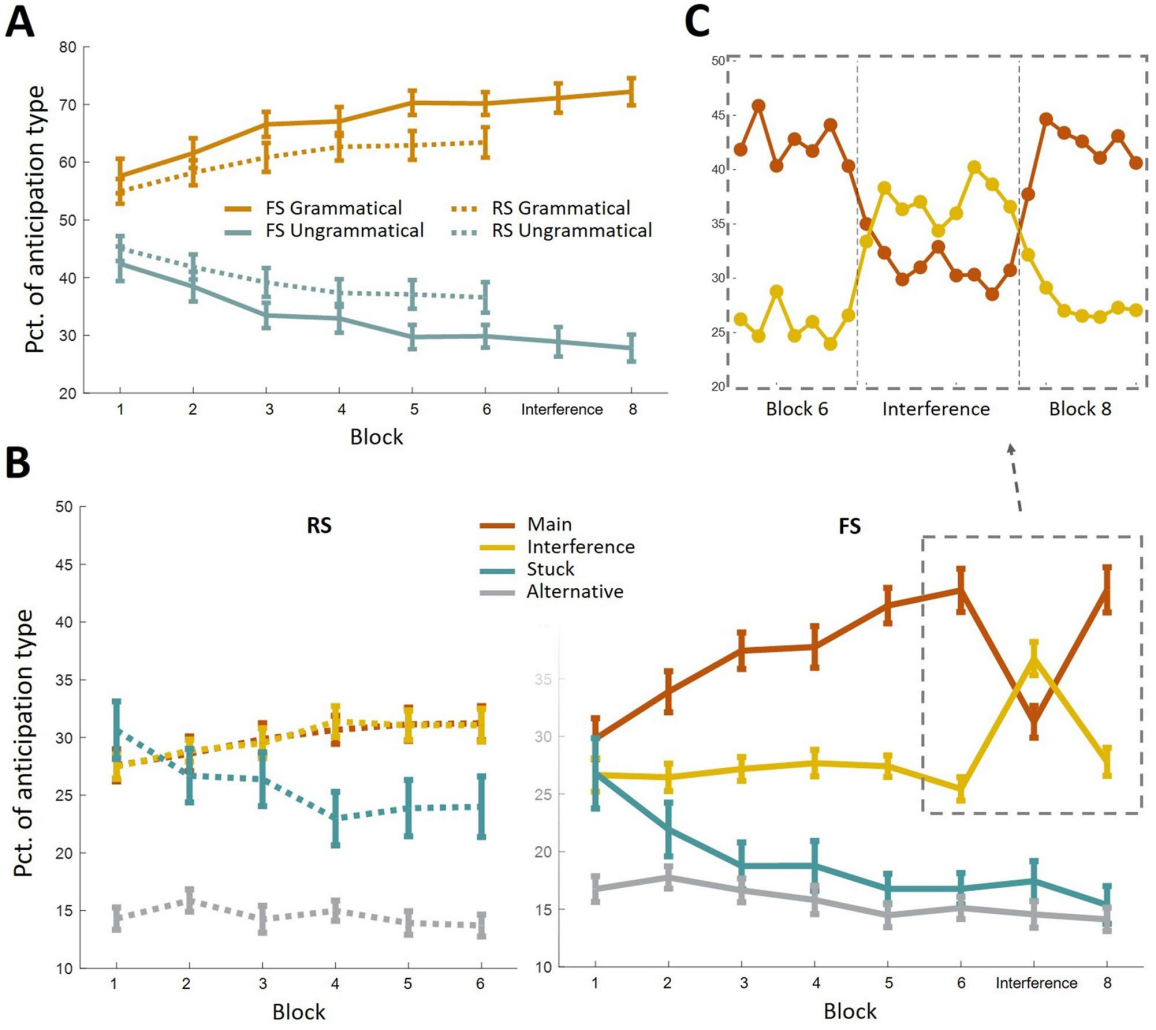
Distribution of anticipation types throughout the experiment. (**A**) Grammatical and ungrammatical fixations evolve similarly over practice in the FS and the RS groups and are not modulated by sequence interference. (**B**) ISI fixations are categorized into anticipation types according to the pair of stimuli that preceded them: *Main* is fixation at the location of the next target according to the sequence governing the task in blocks 1 to 6, *Interference* is at the next target location according to the sequence governing block 7, *Stuck* corresponds to fixation at the location of the last target, and *Alternative* at the location that is neither of the three. Anticipations of the RS group are given on the left panel in dotted lines and of the FS group on the right in solid lines. Error bars denote standard error of the mean. (**C**) Examining Main and Interference anticipations in sequence-by-sequence resolution uncovers quick modulation of behavior following a change in the underlying sequence in the FS group.

Both the FS group and the RS group demonstrated an increase in the rate of grammatical over ungrammatical fixations over the first six learning blocks (*F*(3, 172.9) = 18.97, *p* < 0.001, *η*^2^_*p*_ = 0.25; Fig. 2A). The groups did not differ in this rate or in its increase over time (main effect of group: *F*(1, 57) = 2.99, *p* = 0.09, *η*^2^_*p*_ = 0.05; interaction between group and block: *F*(3, 172.9) = 0.96, *p* = 0.41, *η*^2^_*p*_ = 0.02). This suggests that both groups learned the grammar of the task in a similar manner. A main effect of block existed across all eight blocks of the FS group (*F*(2.9, 81.4) = 13.06, *p* < 0.001, *η*^2^_*p*_ = 0.32). Follow-up analysis revealed that sequence interference did not affect the rate of grammatical fixations (interference effect: *t*(28) = − 0.54, *p* = 0.59; recovery effect: *t*(28) = − 0.76, *p* = 0.46). This is further indication that the knowledge of the task grammar, which is true during the Interference block just as during the Main sequence blocks, is knowledge that participants had acquired separately from knowledge of the particular sequence embedded in the task. We next examine the different types of anticipations participants made, reflecting separate learning of the task sequence and of two grammatical rules.

### Anticipation semantics

Fixations were categorized according to the pair of target locations that preceded them in the stimuli stream (e.g. top and then bottom)^9^. For the FS group, the four target locations correspond to four different types of anticipation. The first type is a fixation at the location that follows that pair in the Main sequence governing the task, hence a grammatical and predominantly correct anticipation (“Main anticipation”; e.g. top–bottom-right). The second type is a fixation at the location following that pair in the Interference sequence (“Interference anticipation”; e.g. top–bottom-left), hence a grammatical anticipation but one that is nevertheless incorrect during the first six blocks. The third type of fixation is at the location of the last target (“Stuck anticipation”; e.g. top–bottom-bottom), which is ungrammatical and always incorrect as there are no repetitions in the task. The last type of fixation is at the remaining fourth location (“Alternative anticipation”), predominately entailing a reversal (e.g. top–bottom-top) which, as mentioned before, is ungrammatical.

For the RS group, there is no distinction between the two grammatical fixations (Main anticipation and Interference anticipation) because there is no sequence governing the task. Because we wish to compare the anticipatory behavior of FS and RS participants, we arbitrarily regard *sequence A* as the Main sequence and *sequence B* as the Interference sequence of the first fifteen RS participants, and the other way around for the last fifteen RS participants (Fig. 2B left panel). The rates of the two types of anticipation were indeed equivalent under this partition (main effect of anticipation type: *F*(1, 29) = 0.004, *p* = 0.95, *η*^2^_*p*_ = 0; interaction between behavior and block: *F*(5, 145) = 0.15, *p* = 0.98, *η*^2^_*p*_ = 0.01). To make sure this equivalence is not a chance result of our arbitrary partition, we conducted 10,000 random partitions of the RS group, in which one half is assigned *sequence A* as the Main sequence and *sequence B* as the Interference sequence and the other half the other way around. Only 1.9% of partitions produced a significant main effect of behavior and 1.8% a significant interaction effect (significance defined as *p* < 0.05).

Following is an analysis of how anticipations distributed across the four different anticipation types. It is important to remember that all anticipations are accounted for at any given time point, and so increase in one type must be accompanied by decrease in another. We therefore can only examine how the distribution changed over time, but cannot infer what particular behavior had driven change.

#### Main anticipation

Main anticipations reflect the sequence learning signal sought after in the SRT task, constituting, in the FS group, the correct anticipation signal in all blocks but the interference block (Fig. 2B red plot). Main anticipations of both groups inclined during the six learning blocks (*F*(3.8, 216.2) = 22.9, *p* < 0.001, *η*^2^_*p*_ = 0.29). However, the rate of Main anticipation in the FS group inclined in a steeper manner (main effect of group: *F*(1, 57) = 15.12, *p* < 0.001, *η*^2^_*p*_ = 0.21; interaction between group and block: *F*(3.8, 216.2) = 6.6, *p* < 0.001, *η*^2^_*p*_ = 0.1), reflecting the effect of sequence learning above and beyond the effect of grammar learning.

Within the FS group, a main effect of block existed across all eight blocks (*F*(4.3, 119.2) = 22.96, *p* < 0.001, *η*^2^_*p*_ = 0.45). A learning effect is evident in the incline of Main anticipations across the six learning blocks (*F*(3.6, 99.6) = 21.98, *p* < 0.001, *η*^2^_*p*_ = 0.44). Interference and recovery effects are evident in the drop and rise of Main anticipation rates in blocks seven and eight respectively (interference effect: *t*(28) = 6.57, *p* < 0.001, recovery effect: *t*(28) = − 6.8, *p* < 0.001). Although this new signal seems to mirror the major effects found in RT, there is only weak correlation between learning effects as measured in Main anticipation and in RT (*r*(27) = − 0.31, *p* = 0.11; see *Statistical analyses* for a description of how learning scores are calculated for correlation analyses). We suggest that this is due to improvement in S-R mapping affecting RT but not the oculomotor signal. This confound is the reason the interference manipulation was introduced into the SRT task to begin with. As already known, learning and interference effects found within RT are not significantly correlated with one another (*r*(27) = − 0.31, *p* = 0.1) and RT during the interference phase does not return to its initial starting point of block one (*t*(28) = 3.75, *p* < 0.001), both indicating that factors unrelated to sequence order are confounded in the learning effect^8^. In Main anticipations, on the other hand, a correlation does exist between learning and interference effects (*r*(27) = − 0.54, *p* < 0.005), and interference does return performance to baseline level (*t*(28) = − 0.8, *p* = 0.43). This suggests that Main anticipations provide a better distilled measure of sequence learning than RT.

#### Interference anticipation

Interference anticipation reflects a grammatical response that is incorrect, except for in block seven (Fig. 2B yellow plot). The RS group produced more Interference anticipations during the six learning blocks than the FS group (*F*(1, 57) = 5.46, *p* < 0.05, *η*^2^_*p*_ = 0.09). This behavior was modulated by block in both groups (*F*(3.7, 208.3) = 2.69, *p* < 0.05, *η*^2^_*p*_ = 0.05) in a similar manner (interaction between group and block: *F*(3.7, 208.3) = 2.06, *p* = 0.09, *η*^2^_*p*_ = 0.04). Within the FS group, Interference anticipation was modulated by block when considering all eight blocks (*F*(4.4, 122.5) = 14.42, *p* < 0.001, *η*^2^_*p*_ = 0.34). However, no learning effect was found (*F*(3.2, 90.7) = 0.9, *p* = 0.45, *η*^2^_*p*_ = 0.03), only interference (*t*(28) = − 6.55, *p* < 0.001) and recovery effects (*t*(28) = 5.53, *p* < 0.001). Notably, interference and recovery effects are in opposite direction of the standard effects of sequence learning—rising in interference and dropping in recovery.

It is a novel finding that Interference anticipation rises in the interference block when it becomes the correct response. It indicates that adaptation to the Interference sequence is already achieved within a single block. Higher resolution analysis reveals that two or three exposures to the sequence order were sufficient to affect behavior: substantially reducing Main anticipations and increasing Interference anticipations when block seven began, and vice versa when block eight began (Fig. 2C). This adaptation was quicker than learning of the Main sequence. Between blocks one and two of the experiment, learning manifested in an average incline of 4.1% (*SD* = 7.2%) in Main anticipations. In comparison, between blocks six and seven, when the Interference sequence was first encountered, Interference anticipations had risen by 11.3% (*SD* = 9.3%; *t*(28) = − 4.43, *p* < 0.001). This discrepancy is another indication that multiple learning processes underlie the task. By block seven, participants have gained knowledge that greatly facilitates learning of sequences that conform to it, allowing quick adaptation to the Interference sequence.

As additional evidence for this claim, the correlation between acquisition of the new sequence in block seven and its loss in block eight (Interference score vs. Recovery score: *r*(27) = − 0.80, *p* < 0.001) is higher than the analogous correlation in Main anticipations reported before. Importantly, the interference sequence is not necessarily learned within this single block. Interference anticipation in block seven could be a result of elimination: it is the only available response after ungrammatical and Main anticipations have been learned to be incorrect. In either case, though, it is clear that knowledge of the grammar facilitates learning of particular sequences abiding to it, and so it seems that a purer measurement of sequence learning could be obtained in the SRT task for a sequence introduced later in the experiment than at its onset. Learning such a sequence will not be confounded with learning of the grammar, and so would be analogous to learning a song after, and not while, learning the language.

The quick adaptation to the Interference sequence shows that sequence interference, the main manipulation used to assess sequence learning in the SRT task, cannot be measured precisely via RT. Sequence interference is designed to capture performance cost. But the cost associated with loss of the original sequence is confounded with new benefit gained from adapting to the new one. The actual cost of sequence interference is therefore greater than what is typically observed. The two effects counteract each other in RT because it reflects only the correctness of anticipation and not its content.

The acquisition of background knowledge that facilitates learning had been termed, among other names, "learning sets" in psychology^10^, and “inductive biases”^11^ or “meta-learning”^12^ in computational learning algorithms. It is paramount in real-life learning, albeit under-regarded in laboratory settings. We suggest that in block seven, the background knowledge, i.e. the task grammar, "is the mechanism that changes the problem from an intellectual tribulation into an intellectual triviality and leaves the organism free to attack problems of another hierarchy of difficulty"^10^.

#### Alternative anticipation

The Alternative anticipation was performed at a similar rate (*F*(1, 57) = 1.84, *p* = 0.18, *η*^2^_*p*_ = 0.03) and decreased similarly across learning blocks in both the FS and the RS groups (main effect of block: *F*(5, 285) = 3.96, *p* < 0.005, *η*^2^_*p*_ = 0.07; interaction between group and block: *F*(5, 285) = 0.72, *p* = 0.61, *η*^2^_*p*_ = 0.01; Fig. 2B gray plot). Within the FS group, a main effect of block existed across all eight experimental blocks (*F*(7, 196) = 3.56, *p* < 0.005, *η*^2^_*p*_ = 0.11). A learning effect existed across the learning blocks (*F*(5, 140) = 2.8, *p* < 0.05, *η*^2^_*p*_ = 0.09), but no interference (*t*(28) = 0.61, *p* = 0.55) or recovery (*t*(28) = 0.49, *p* = 0.63) effects were found. Alternative anticipations therefore reflect a learning process that is not affected by sequence change, consistent with our claim that participants learn the task grammar in the SRT task separately from the task sequence.

Following 11 out of the 12 stimuli-pair “prefixes” existing in the current task, the alternative location corresponds to the location of the first element of the pair. This means that fixating on the alternative location requires returning to the location before last (e.g. a fixation at the top location following targets at the top and then at the right). This back-and-forth response is a-priori unnatural due to inhibition of return^13^. However, because such reversals have been implicated before in RT facilitation due to their saliency^14^, we believe the opposite effect found here is a reflection of acquired grammar and not of inherent tendencies. This is evident in the contrast between Alternative and Interference anticipations in the FS group. Both behaviors, in the first six blocks, constitute a shift in gaze to an incorrect new location. However, the grammatical mistake (Interference anticipations) is more commonly made than the ungrammatical one (Alternative anticipations; *F*(1,28) = 69.25, *p* < 0.001, *η*^2^_*p*_ = 0.71), even though neither transition has been encountered before and they are just as wrong.

The Alternative anticipation was also the least prevalent of all anticipation types. In the first block already, while all other anticipations happened at a similar rate in the FS group (as would be expected prior to learning), Alternative anticipations happened significantly less (evident in the effect of anticipation type in block one *F*(1.4, 40.1) = 6.16, *p* = 0.01, *η*^2^_*p*_ = 0.18, an effect that is gone when excluding Alternative anticipations *F*(1.2, 33.4) = 0.45, *p* = 0.54, *η*^2^_*p*_ = 0.02). This suggests that the rule rendering this anticipation type ungrammatical has been learned very early in the experiment, within the first block. Such learning could be accomplished by statistical learning, a faculty enabling quick extraction of regularities embedded in the environment^15^. Statistical learning had been proposed in the literature to facilitate numerous learning processes^16^ including sequence learning^14,17^, and can occur within few exposures to a regularity^18^. The learning expressed in Alternative anticipations therefore reflects statistical learning of a second order transition rule (i.e. relating to the last two targets).

#### Stuck anticipation

Although overall more prevalent in the RS group (*F*(1, 57) = 4.2, *p* < 0.05, *η*^2^_*p*_ = 0.07), rates of Stuck anticipations steadily drop throughout the learning blocks in both groups (*F*(2.8, 159.4) = 14.51, *p* < 0.001, *η*^2^_*p*_ = 0.2), in a similar manner (interaction between block and group: *F*(2.8, 159.4) = 0.99, *p* = 0.39, *η*^2^_*p*_ = 0.02; Fig. 2B turquoise plot). As with Alternative anticipations, this drop reflects statistical learning. Here, though, this learning relates to a first order transition rule, that consecutive targets do not occupy the same location. Indeed, within the FS group (main effect over all eight blocks: *F*(2.6, 73) = 8.57, *p* < 0.001, *η*^2^_*p*_ = 0.23), this learning is also unaffected by sequence shift in block seven (interference effect: *t*(28) = − 0.51, *p* = 0.61, recovery effect: *t*(28) = 1.69, *p* = 0.1), indicating that it is independent of the particular sequence governing the task. However, learning of Stuck anticipations is markedly slower and more gradual than that reflected in Alternative anticipations, even though it relates to a statistical rule that is more noticeable and more consistent. Also, it is the only oculomotor signal in which learning correlates with learning of Main anticipation (*r(*27*)* = − 0.68, *p* < 0.001), suggesting it has a more central role than others in the task’s sequence learning. We suggest that in addition to statistical learning, Stuck anticipation captures a higher-scale strategy in the task.

A fixation on Stuck location is not only a guess of where the next target will appear, but also a response that is essentially different than fixations on other locations: it is not putting effort in an anticipation attempt. Such behavior may be due to lesser engagement in the task or may reflect a (non-mutually exclusive) process of “learning to try”. While participants do not have reasonable confidence that there is a regularity governing where targets appear, withholding anticipation attempts is perfectly reasonable. Even more so, as demonstrated in the next section, a wrong anticipation attempt is costly in RT compared to no anticipation attempt, so no attempt may be preferable when chances are against succeeding in it. The decline in Stuck anticipations may therefore reflect learning that a regularity governs the task (regardless of what that regularity is), and so that trying to guess the next target location is a worthwhile effort and leaving gaze in place is not. Indeed, it has been shown that implicit statistical learning is sensitive to the mere existence of a regularity in the environment^19^. This may also account for the higher rate of Stuck anticipations in the RS group compared to the FS group. For RS participants, to leave gaze in place was a better strategy than for FS participants, either because the chance of failing to anticipate targets was always greater than the chance of succeeding, or simply because the FS group also had sequence learning to guide behavior and the RS group did not.

**Figure 3:**
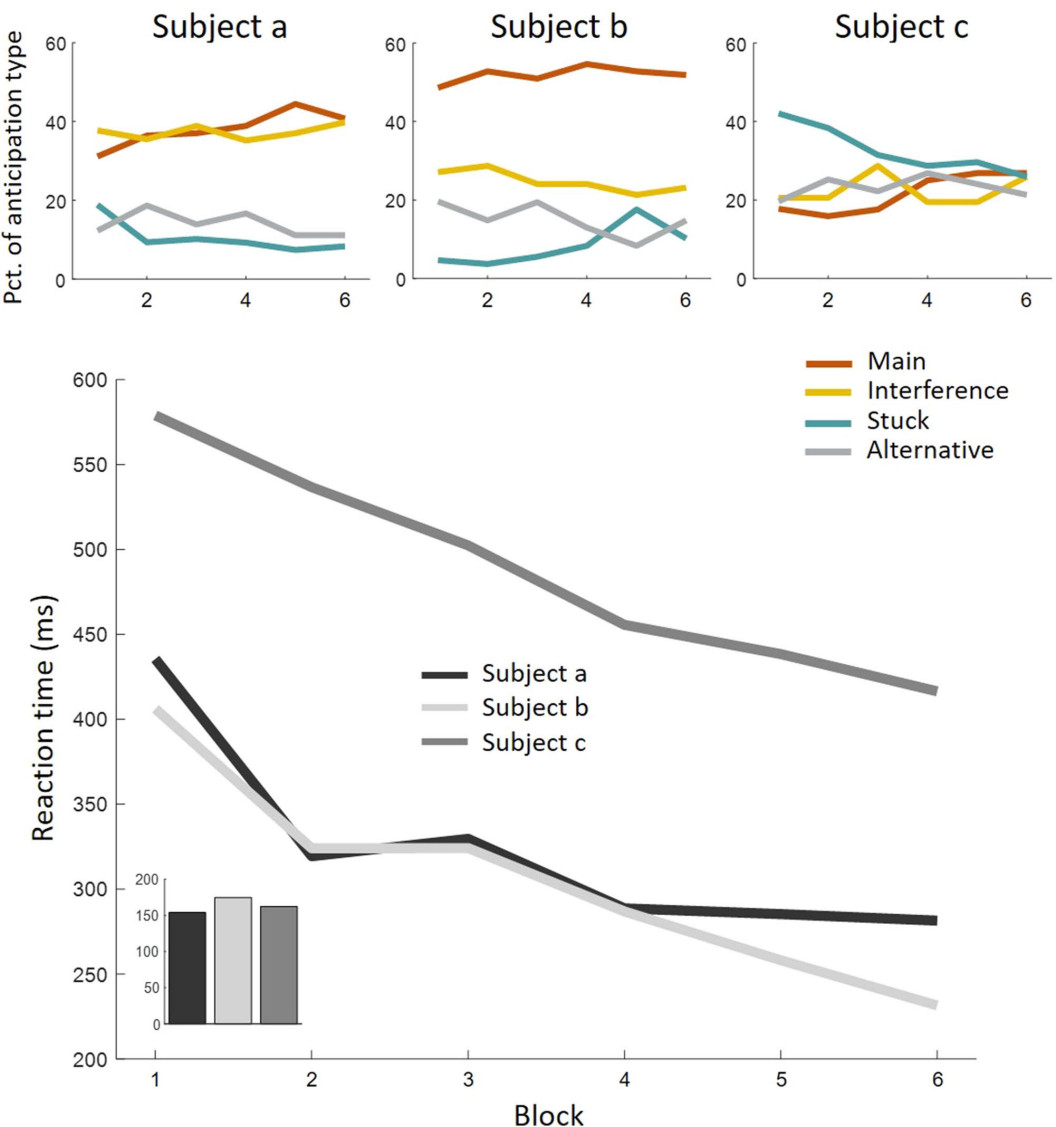
Different underlying processes producing similar learning effects in RT. **Top panel:** Oculomotor behavior of three participants of the FS group during blocks 1–6. A standard learning effect can be seen in *subject a*'s oculomotor behavior, almost no learning effect in *subject b*'s oculomotor behavior, and mostly Stuck learning in *subject c*'s oculomotor behavior. **Bottom panel:** RT of the same participants in blocks 1–6. A similar learning effect in RT was achieved by the three participants, even though at different overall speeds. Aggregated learning effect (block 6 minus block 1) given in inset figure. The similar RT effect was achieved for predominantly different reasons: via more anticipation of targets, via better anticipation of targets, and via release from Stuck, for participants *a*, *b* & *c,* respectively.

Interestingly, Stuck learning correlated with RT learning in the FS group (*r*(27) = 0.52, *p* < 0.005), while, as described before, the correlation of Main anticipation learning with RT learning was only marginal. This implies that the major driving force of the standard RT effect found in the SRT task may not be learning of the sequence order, but rather the pre-requisite of attempting to learn. A tendency to remain stuck in place and the ability to release from it may be cognitive attributes important to performance in the SRT task but orthogonal to sequence learning. As demonstrated in Fig. 3, participants in our study had very different styles of oculomotor anticipatory behavior yielding similar RT effects, nonetheless. Studies in recent years have demonstrated individual differences in statistical learning^20^, that may be due to different learning strategies^21^. Whether a transient tendency or a stable trait, withholding or engaging in anticipation attempts may be seen as more or less conservative strategies on the exploration–exploitation dilemma in learning^22^. Sequence learning may be tightly coupled to mere attempts of an anticipation simply because an error is required to drive error-based learning. Future study is needed to affirm this hypothesis, and to examine whether different strategies of anticipation correlate with known decision-making mechanisms and deficiencies.

This insight has particularly strong implications for usage of the SRT task in clinical research. RT discrepancies between populations are usually taken to indicate impairments in different learning mechanisms, but the current findings imply that RT similarities between populations (e.g. amnesics^4,23^, Alzheimer's disease^24^ and normal elderly^25,26^) may nevertheless conceal different underlying processes. Further research is needed to dissociate sequence learning from "Stuck learning" and other types of statistical learning both in individuals and in clinical populations. In Schwizer Ashkenazi, Sacher and Vakil (2020)^27^, for instance, we have found that impaired implicit sequence learning in the O-SRT task by individuals with traumatic brain injury stemmed from their higher rate of Stuck anticipations.

### Motor response under different anticipations

**Figure 4:**
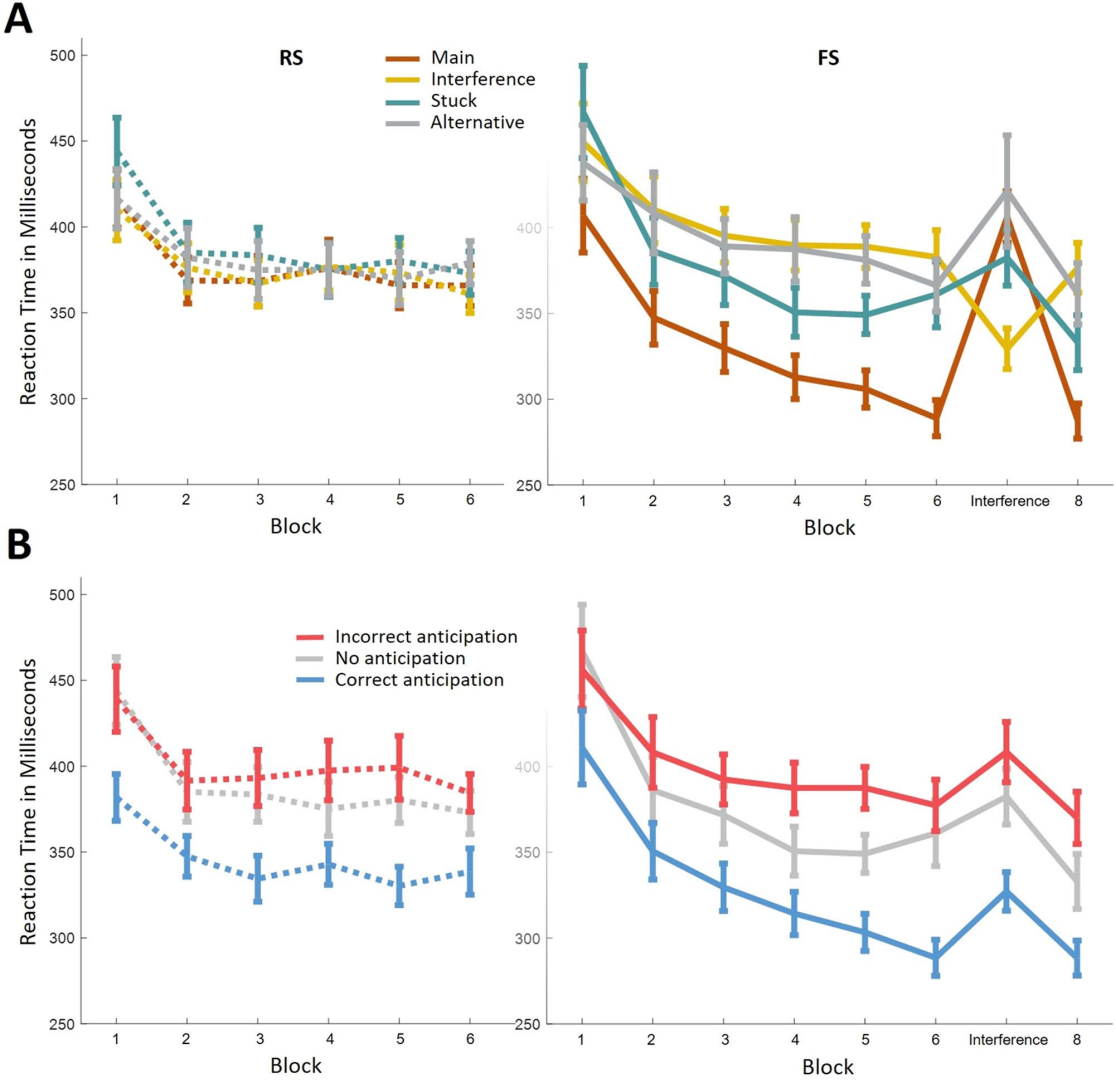
RT according to the oculomotor anticipation preceding it. (**A**) RT in all trials improve with learning. RT of the RS group is similar across all types of anticipation preceding the trial (Left panel). RT of the FS group shows distinct modulation by anticipation type. Namely, RT following Main anticipations have the steepest improvement curve. During the learning phase (blocks 1 to 6) RT following Main anticipations (correct anticipations) is quickest, RT following interference or Alternative anticipations (incorrect anticipations) is the slowest and RT following Stuck anticipations (no anticipation attempt) is in between. (**B**) Data of (**A**) is depicted according to the correctness of the anticipation attempt preceding the trial. In the RS group, correct anticipation yields quicker response, as would be expected. This difference was not evident in (**A**) because Main and Interference anticipations are both a mix of correct and incorrect anticipations. In the FS group, it should be noticed that during sequence interference (block seven) Main anticipations are incorrect and Interference anticipations are the new correct anticipation. This portrayal of the data from (**A**) demonstrates that sequence interference has a similar effect on all trials, regardless of anticipation correctness. Error bars denote standard error of the mean.

We end this report by returning to the traditional RT signal and re-analyzing it in light of our new understanding of the oculomotor signal. While the results presented thus far may seem to suggest that oculomotor measures completely explain-away RT data, this is not the case. Parsing RT according to the oculomotor signal uncovers sequence learning effects that were not observed in the oculomotor signal alone (Fig. 4A).

ANOVA over RT of the FS group considering all eight blocks and all anticipation types as within-subject factors, revealed modulation by both (main effect of block: *F*(3.4, 96.5) = 13.47, *p* < 0.001, *η*^2^_*p*_ = 0.33; main effect of anticipation type: *F*(1.4, 39.4) = 40.03, *p* < 0.001, *η*^2^_*p*_ = 0.59; interaction between block and anticipation type: *F*(6.5, 181.7) = 9.67, *p* < 0.001, *η*^2^_*p*_ = 0.26). A main effect of block across the learning blocks and regardless of anticipation type (*F*(2.7, 75.6) = 15.59, *p* < 0.001, *η*^2^_*p*_ = 0.36) is expected due to improvement in S-R mapping. However, an interaction exists between block and anticipation type also within these six blocks (*F*(6.7, 186.7) = 3.46, *p* < 0.005, *η*^2^_*p*_ = 0.11). Follow-up simple effects tests reveal that RT following Main and Stuck anticipations share a different trajectory than RT following Interference and Alternative anticipations (interaction of Main and Stuck anticipation types with block: *F*(3.2, 88.4) = 1.87, *p* = 0.14, *η*^2^_*p*_ = 0.06; interaction of Interference and Alternative anticipation types with block: *F*(3.6, 100.7) = 0.37, *p* = 0.81, *η*^2^_*p*_ = 0.01). This division reflects the effect of anticipation correctness, and its gradual refinement. Interference and Alternative anticipations are equally incorrect and accordingly yield the same modest improvement in RT that can be attributed to S-R mapping. The steeper curves of Main and Stuck anticipations reflect learning of the stimuli structure.

Under Stuck anticipations, RT improvement that is greater than that afforded by S-R mapping could be due to covert attention^3,28^. Such anticipation, that is not expressed in eye movement, would be missed by our oculomotor analysis, but would nevertheless facilitate RT if it were towards the correct location. As previously discussed, a strategy of leaving gaze in place and covertly learning the task structure is entirely viable.

RT under Main anticipation also improves beyond S-R mapping, even though in these trials gaze is always in the correct location when the target appears. This could be explained by two factors. First, as learning progresses, more correct anticipations occur on purpose in opposed to by chance. Motor preparation would accompany purposeful anticipation but not necessarily chance anticipation, and so the former would yield quicker RT than the latter. Second, even under correct and purposeful anticipation, response can be gradually refined by increased practice and confidence in the anticipation.

Collapsing anticipation attempts into “correct”, “incorrect” or “no” attempt of anticipation provides further insight into the dissociation between sequence learning and S-R mapping (Fig. 4B). RT following correct anticipations in the first six blocks of both the FS and the RS groups is similar (*F*(1, 57) = 0.59, *p* = 0.45, *η*^2^_*p*_ = 0.01) and modulated by block (*F*(3.2, 179.9) = 35.75, *p* < 0.001, *η*^2^_*p*_ = 0.39). However, an interaction exists between group and block (*F*(3.2, 179.9) = 7.45, *p* < 0.001, *η*^2^_*p*_ = 0.12). RT following correct anticipation in the FS group improves more steeply than in the RS group. We attribute this to more purposeful anticipations and more refined responses in the FS group, as described above, because correct anticipations in the RS group can be made with no more than 50% chance.

Lastly, RT during sequence interference affords one addition finding. No interaction is found between anticipation correctness (correct, incorrect or no anticipation attempt) and RT Interference effect (*F*(1.4, 39.2) = 1.25, *p* = 0.29, *η*^2^_*p*_ = 0.04). In other words, sequence change delayed RT in trials of correct anticipation, no attempt of anticipation, and, most notably, incorrect anticipation, to the same extent. The existence of a new regularity seems to take a toll on response speed regardless of the ease to respond in any particular trial. It could be that the appearance of a new sequence order taxes resources for adaptation and learning, processes that extend beyond individual trials. Alternatively, performance cost may be due to the predictability of task stimuli. The introduction of the new sequence undermines the validity of all stimuli that were used as predictors beforehand. Vaskevich and Luria (2018)^29^ suggest that this promotes re-evaluation of the reliance on prior information, leading to an overall reduction in response speed across all trials, as is indeed observed here. RT, therefore, indicates not only how well the current sequence is anticipated, but also how volatile it is perceived to be, and this evaluation is an additional process involved in successful sequence learning.

## Conclusion

In this report we demonstrate that oculomotor behavior, put in the context of the sequence driving the SRT task, sheds light on what is learned throughout the task. We find that three major learning processes take place during the task somewhat independently: (1) learning to try to anticipate the sequence (2) learning constraints on what to anticipate in the sequence (3) learning to anticipate the sequence. To our knowledge, this is the first evidence of (1) in the SRT literature, despite its substantial influence on performance, and the first demonstration of the interplay between (2) and (3), evidence for which has so far been indirect. Overall, we show that underlying the seemingly simple SRT task are tightly-woven intricate learning processes^30^. We find this encouraging, as higher-resolution dissociations could now be made between processes reflected in brain activity, between individuals in psychological studies, and between populations in clinical studies. In our opinion, the combination of motor with oculomotor signals provides the best method to achieve this to date.

## Methods

### Participants

Fifty-nine undergraduate students participated in the experiment for course credit or 30 NIS (roughly 10 USD). Participants comprised 22 males and 37 females, at a mean age of 24.7 years (range: 18–37 years). The FS group comprised twenty-nine participants who constituted the “MA group” in the experiment published in Vakil et al. (2007)^8^. The RS group comprised thirty additional participants. Data of both groups had also been used in Tal and Vakil (2020)^9^ to examine learning of individual elements within the SRT task. The study was approved by the ethics committee of the Psychology Department in Bar-Ilan University. All research was performed in accordance with relevant guidelines and regulations and each participant gave written informed consent.

### Stimuli

Four white squares, in diamond formation, were presented against grey background on an LCD computer screen of 1680 X 1050 pixel resolution (size 47 X 29 cm). A black dot indicating the target could appear in the center of one of the squares, at a visual angle of 8.8° from the screen center, or not appear in any. Squares were of size 6 × 6 cm, and dots of 1.5 cm diameter.

### Procedure

Participants were seated 60 cm in front of a computer screen. They were asked that when targets (dots) appear on screen they locate them in their vision as soon as possible and press a corresponding keyboard key, according to their location: up arrow for the top square, left arrow for the left square, etc. Each target appearance lasted 3000 ms or until a button was pressed. When targets disappeared, no target was displayed on screen for an ISI of 500 ms, after which the next target appeared.

In the FS group, unbeknownst to participants, target locations followed a fixed sequential order of length 12. As prevalently used in the SRT task, sequence order was second-order conditional (SOC). In SOC sequences target locations do not repeat back-to-back, and both location frequency and first-order transition probabilities are counter-balanced^14^. Two such sequences were used, *sequence A*: 3–4–2–3–1–2–1–4–3–2–4–1 and *sequence B*: 3–4–1–2–4–3–1–4–2–1–3–2 (numbers corresponding to location: 1-down, 2-left, 3-right, 4-up). Nine concatenated sequences constituted one block (108 stimuli). The FS experiment consisted of 8 blocks in total, each starting from a different position within the sequence: locations 1, 5, 10, 8, 4, 12, 1, 2 for blocks 1–8 respectively. Blocks 1–6 and block 8 were constructed from one sequence (the "Main sequence"), while block 7, called the Interference block, was constructed from the other (the "Interference sequence"). FS participants were randomly assigned into those whose Main sequence was *sequence A* and their Interference sequence was *sequence B*, and those who had it the other way around (*n* = 15 & *n* = 14 respectively).

In the RS experiment the task was the same but no fixed sequence guided target locations. Instead, the order of target locations maintained the statistical characteristics of the FS stimuli stream while no repeating sequence of locations inhabited it. This was done so that task grammar would be equivalent in both experiments, even though sequence learning is possible only the FS group. Therefore, all locations in the RS task appeared at near uniform frequency (*M* = 25% *SD* = 0.3%). Locations did not repeat back-to-back, but all other first-order transitions (e.g. 1, 3 or 4 after 2) appeared at near uniform frequency (*M* = 33.3% *SD* = 0.9%). Also, twelve triplets that constitute a reversal (e.g. 2–1–2, 2–3–2, 2–4–2, …) all occurred at a similar frequency (*M* = 1% *SD* = 0.1%), comprising together 11.7% of the stimuli stream, which is slightly higher than the 8.8% they constitute in the FS stimuli stream (due to one reversal that inhabits that order). Lastly, all other second-order transitions (e.g. 3 or 4 after 2–1) appeared at near uniform frequency (*M* = 44.1% *SD* = 1.4%). As in the FS task, participants were unaware of any structure guiding target locations in their task. As there was no fixed sequence underlying target locations in the RS task, interference and recovery blocks are irrelevant, and the RS experiment was of six blocks only (648 targets).

### Data acquisition

Participants' keypress times and eye movements were collected throughout the experiment. Eye movements were captured using SMI iView 250 RED Eye Tracker. Calibration was done at experiment onset using a standard 9-point grid for both eyes.

### Response time analysis

RT corresponds to the time since stimuli appeared and until the correct key was pressed. Trials of incorrect or no response were removed from analysis (0.8% of the data).

### Oculomotor anticipation analysis

Fixation analysis was done for eye movements captured during ISIs. Fixations were found in 98.8% of ISIs. ISIs were subsequently categorized according to which of the four target locations their fixation was closest to (effectively dividing the screen into four triangular regions surrounding each target location^8^; Fig. 1C). Thus, a categorical variable was formed, indicating what regions were fixated on before upcoming targets, and interpreted as where participants anticipated the next target to appear. In 54.8% of ISIs a fixation was detected in more than one of these regions. In 91.6% of these multiple-region ISIs, the first fixation was on the previous target region and so most likely represents carryover from the previous trial rather than anticipation of the next (Fig. 1C). Therefore, when there was more than one fixated region during an ISI, only the last fixated region was considered.

In SOC sequences, target locations are completely determined by the two locations preceding them. Therefore, the four possible fixation locations can be categorized in the FS experiment into four different “semantic” locations according to the two preceding targets and the sequence order. Given the last two targets, a fixation during an ISI can either be at the location that would be expected next according to the Main sequence ("Main anticipation"), at the location that would be expected next according to the Interference sequence (even though the interference sequence is encountered only in block seven; "Interference anticipation"), at the other location that is neither of these two ("Alternative anticipation"), or simply at the same location that the last target appeared in ("Stuck anticipation", because it corresponds to leaving gaze in place)^9^.

The requirement of two preceding targets deems ISIs at experiment onset, and at certain transitions between blocks which produce neighboring targets that do not appear in the experimental sequences, irrelevant for this categorization (except for Stuck anticipation that can be determined based on one preceding target only). After removing unusable ISIs and missing trials, analysis encompassed 98.6% of ISIs.

### Statistical analyses

Behavior is analyzed using repeated measures ANOVA across all experimental blocks. When examining the FS group alone this entails eight blocks, but when comparing FS to RS groups this entails using only the first six blocks of the FS group (the learning blocks). Then, post-hoc analyses are carried out on FS data. Learning effect is tested via repeated measures ANOVA over the first six learning blocks. Interference effect is tested via t-test contrasting block six with block seven, and recovery effect is tested via t-test contrasting block seven with block eight. All t-tests reported in this study are paired t-test, two-tailed. For correlation analyses, a single score is calculated for each effect. The learning score is calculated by subtracting performance in block six from performance in block one, Interference score by subtracting performance in block seven from performance in block six, and Recovery score by subtracting performance in block eight from performance in block seven. For consistency across measures, all contrasts between blocks are coded in chronological order, such that the value of the later block is subtracted from the value of the earlier block. In cases in which Mauchly’s test of sphericity was significant (*p* < 0.05), Greenhouse–Geisser corrected values are reported.

## Data Availability

The data used for this work are available at https://osf.io/rj692/.

## Code availability

The custom code used in this study is available from the corresponding author upon reasonable request.
